# The E3 ligase MuRF2 plays a key role in the functional capacity of skeletal muscle fibroblasts

**DOI:** 10.1590/1414-431X20198551

**Published:** 2019-08-29

**Authors:** J.G. Silvestre, I.L. Baptista, W.J. Silva, A. Cruz, M.T. Silva, E.H. Miyabara, S. Labeit, A.S. Moriscot

**Affiliations:** 1 Departamento de Anatomia, Instituto de Ciências Biomédicas, Universidade de São Paulo, São Paulo, SP, Brasil; 2 Faculdade de Ciências Aplicadas, UNICAMP, Limeira, SP, Brasil; 3 Institute for Integrative Pathophysiology, Mannheim Medical University, Faculty for Clinical Medicine Mannheim, University of Heidelberg, Mannheim, Germany

**Keywords:** Fibroblast, Skeletal muscle, Migration, MuRF2, E3 ligase, Wound healing

## Abstract

Fibroblasts are a highly heterogeneous population of cells, being found in a large number of different tissues. These cells produce the extracellular matrix, which is essential to preserve structural integrity of connective tissues. Fibroblasts are frequently engaged in migration and remodeling, exerting traction forces in the extracellular matrix, which is crucial for matrix deposition and wound healing. In addition, previous studies performed on primary myoblasts suggest that the E3 ligase MuRF2 might function as a cytoskeleton adaptor. Here, we hypothesized that MuRF2 also plays a functional role in skeletal muscle fibroblasts. We found that skeletal muscle fibroblasts express MuRF2 and its siRNA knock-down promoted decreased fibroblast migration, cell border accumulation of polymerized actin, and down-regulation of the phospho-Akt expression. Our results indicated that MuRF2 was necessary to maintain the actin cytoskeleton functionality in skeletal muscle fibroblasts via Akt activity and exerted an important role in extracellular matrix remodeling in the skeletal muscle tissue.

## Introduction

Mesenchymal cells are known for giving rise to a large number of connective tissue cells such as myoblasts, chondroblasts, adipocytes, osteoblasts, and fibroblasts. Fibroblasts, in turn, are a highly heterogeneous population of cells, and can be found in different tissues (1), being the most abundant stromal cell type (2). The extracellular matrix (ECM), produced by fibroblasts, plays essential roles in preserving the structural integrity of connective tissues and during healing progression (1). In skeletal muscle tissue, fibroblasts are one of the most abundant cell population (3). The ECM is an important scaffold for skeletal muscle fibers and is involved in force transmission and protection against mechanical trauma (4).

Regeneration of skeletal muscle is a highly complex process that involves the activation of a large number of intracellular and cell-to-cell responses (5,6). Although it is widely known that the regeneration process relies upon satellite cells in order to repair the skeletal muscle fiber and recover function, the much less understood fibroblasts also play an important role. Recently, there has been increasing evidence of a role for the ECM in signaling through regulatory molecules (7). Interestingly, however, it is known that excessive ECM can impair muscle function and hinder muscle regeneration (8,9). In wound repair, fibroblasts proliferate and differentiate into active states, secreting important components of the ECM. These cells also intensely engage in migration and remodeling and exert traction forces in the ECM (10).

The migratory capacity of fibroblasts is crucial for matrix deposition and wound healing (11), and recent studies highlight the importance of correct cytoskeleton arrangement for fibroblast migration. Noteworthily, it has been shown that Akt activity is crucial for the establishment of actin filaments at the edge of fibroblasts during the migration process (12). Recently, it has been shown that E3 ubiquitin ligases play important roles in cytoskeleton dynamics towards proper migration (13).

The E3 ubiquitin ligases are proteins that play essential roles in cellular homeostasis and in the degradation of mutated misfolded or damaged proteins (14,15). In skeletal muscle tissue, an important family of E3 ubiquitin ligases initially named muscle-specific RING finger (MuRFs) has been described (16). Subsequently, MuRFs were categorized as MuRF1 (17) and MuRF2 (18). MuRF2 can establish direct binding with stable microtubules and proteins at the M-line region, and the silencing of MuRF1 and MuRF2 disrupts the structure of microtubules and intermediate filaments (18).

Thus, due to the common progenitor shared by fibroblasts and skeletal muscle cells, the large number of fibroblasts present in skeletal muscle niche, the importance of cytoskeleton organization for fibroblasts migration, and the role of MuRF2 in the stabilization of microtubules, our study aimed to test whether MuRF2 is expressed and if it plays a functional role in skeletal muscle fibroblasts.

## Material and Methods

The protocols employed in this study followed the ethical guidelines in animal research (Brazilian College of Animal Experimentation). These protocols were submitted and approved by the Institute of Biomedical Sciences/University of São Paulo - Ethical Committee for Animal Research (# 118/13).

### Fibroblasts and myogenic cell culture

Purified cells were obtained from primary culture using a modified version of a previously described preplating technique (19). Cells were isolated from the hind limb muscles of male FVB mice (∼8 weeks old, 30±3 g). Cell isolation and digestion were made as previously described (20,21). After digestion, the cells were incubated at 37°C in an atmosphere of 5% CO_2_ for 40-50 min on 75 cm^2^ tissue culture dishes. The supernatant was used as myoblast-enriched primary skeletal muscle culture (see below) and the rapidly adhering fibroblasts were maintained (P0) as previously reported (19). These fibroblast populations were kept up to 80-90% confluence and then passed using the same protocol described above, always discarding the non-adherent supernatant cells. This procedure was repeated 3 times. All experiments using fibroblasts were performed at the third passage (P3).

Both C2C12 (ATCC CRL-1772, USA) and the supernatant cells from P0 myoblast-enriched pool were plated in Matrigel-coated 24-well plates (Matrigel Matrix Growth Factor Reduced; BD Biosciences #354230, USA) and incubated until 2 days after differentiation as previously described (20,21).

### Cardiac fibroblasts

Primary cardiac fibroblasts were obtained as previously described (22) and kindly provided by Dr. Maria Luiza Morais Barreto-Chaves (Department of Anatomy, University of São Paulo, Brazil).

### Mouse embryonic fibroblasts

Mouse embryonic fibroblasts (MEFs, ATCC#SCRC-1008) were kindly provided by Prof. Dr. Julio Cesar Batista Ferreira (Department of Anatomy, University of São Paulo, Brazil). These cells were maintained in DMEM supplemented with 10% FBS until ∼90% confluency, and subsequently MuRF2 immunofluorescence was carried out.

### Pancreatic islets

Pancreatic islets were kindly provided by Angelo R. Carpinelli (Department of Physiology and Biophysics, University of São Paulo, Brazil). Islets were obtained from Wistar rats as previously described (23).

### siRNA silencing

For small interfering RNA (siRNA) interference assays, we transfected fibroblasts at 80-90% confluence with scrambled (#AM4613, Ambion, USA) and MuRF2 (cat #AM4390771, Ambion) siRNAs. Fibroblasts plated on 24-well plates were transfected with 30 nM siRNAs using siPORT^TM^ NeoFX^TM^ transfection agent (Ambion #AM4511) according to the manufacturer's instructions and as previously described (20). The cells were transfected for 12 h, and then the siRNA solution was replaced with DMEM containing 10% FBS.

### Wound healing assay

Wound healing assays were performed based on a previously described method (24). *In vitro* scratch wounds were created 12 h after siRNA silencing by applying a straight cut on confluent cell monolayers with a sterile disposable pipette tip. Four independent experiments were performed.

### Western blot analysis

Cells were collected and protein lysates were prepared for western blot analysis as previously described (20).

Primary antibodies used were: MuRF2 (18); Akt (Cell Signaling #9272, USA); GAPDH (Cell Signaling #2118); pAkt Ser473 (Cell Signaling #4058); pAkt Thr308 (Cell Signaling #4056). Secondary antibodies used were: Goat anti-rabbit (Jackson ImmunoResearch #111-035-003, USA). After the membranes were washed, specific bands were visualized with the Fusion Fx (Vilber Lourmat Biosystems, France) system after the use of the sensitive chemiluminescent substrate Lumina^TM^ Forte Western HRP Substrate (Millipore, USA). Loading variations were monitored by Ponceau staining and GAPDH. The relative protein expression intensities were quantified by densitometry using ImageJ (1.48 version, Wayne Rasband, National Institutes of Health, USA).

### Immunofluorescence

Cells on a 24-well plate were fixed with 4% paraformaldehyde for 10 min at room temperature, washed with PBS containing 0.1% Triton X-100 (PBS-T) two times for 3 min each and then blocked/permeabilized with PBS-T containing 1% BSA for 1 h. Then, the wells were prepared for immunofluorescence analysis as previously described (20). Fluorescence micrographs were obtained with the microscope Zeiss Imager M1 (Carl Zeiss Microscopy GmbH, Germany) equipped with FITC, rhodamine, and FURA filters. Primary antibodies used were: MuRF2 (18), MyoD (sc-31940), Ki-67 (NB110-89717), and TCF-4 (sc-13027). Secondary antibodies used were: Cy3 Donkey Anti-rabbit (Jackson ImmunoResearch #711-165-152), Cy3 Donkey Anti-goat (Jackson ImmunoResearch #705-175-147), and Cy2 Goat Anti-rabbit (Jackson ImmunoResearch #111-225-008). Digital images from a Zeiss Axiocam MRn were exported as TIFF files with Axiovision 4.6 software without any subsequent processing in parameters such as contrast and brightness.

### Proliferation index

This assay was performed following the same protocol used in the wound healing assay. The proliferation index is reported as the percentage of Ki67-labeled cells (red)/DAPI-labeled cells (blue) over the total number of cells in five fields/well (two wells per group).

### Detection of polymerized actin-containing lamellipodia

This assay was performed 24 h after the onset of wound healing assays and following the same specification used in immunofluorescence analysis using phalloidin-Alexa Fluor 488 (A12379, Invitrogen, USA). The number of positive cells is reported as the percentage of polymerized actin-containing lamellipodia (densely green at the edge of the cell)/DAPI-labeled cells (blue) over the total number of cells in six fields/well (two wells per group).

### Quantitative polymerase chain reaction and semi-quantitative PCR

Cells and tissues were homogenized, and total RNA was obtained using TRIzol reagent (Invitrogen) following the manufacturer's instructions. One microgram of total RNA was used and cDNA was synthesized by cDNA Promega kit (M-MLV-Promega, USA). For semi-quantitative PCR, we used 50 ng of cDNA, amplified by 40 cycles of PCR using Taq DNA polymerase (Sinapse Inc, Brazil).

Real-Time PCR was performed separately for each gene in duplicates in a 25-µL reaction volume (20 ng cDNA, 12.5 µL Sybr Green Master Mix^©^, Applied Biosystems, USA) and 300 nM of each primer. Primer sequences were as follows: MuRF1 forward, CCTGCTGGTGGAAAACATCA, and MuRF1 reverse GGCTATTCTCCTTGGTCACTCT; MuRF2 forward, GTCCTGGTGACACAGATTGGAT, and MuRF2 reverse, TGCTGCCTATGTGCTTCTCA; collagen 1 forward, CTTGGTGGTTTTGTATTCGATGAC, collagen 1 reverse, GCGAAGGCAACAGTCGCT; cyclophilin forward, GCCGATGACGAGCCCTTG, and cyclophilin reverse, TGCCGCCAGTGCCATTAT. Cyclophilin expression served as an internal control and relative levels of mRNA expression were normalized to its expression.

### Statistical analysis

The data are reported as means±SE from three independent experiments. Statistical analysis was performed by Student's unpaired *t*-test between groups using GraphPad Prism v.7 (GraphPad Software, USA). For all comparisons, P<0.05 was considered significant.

## Results

### Validation of fibroblast culture

**Figure 1.**
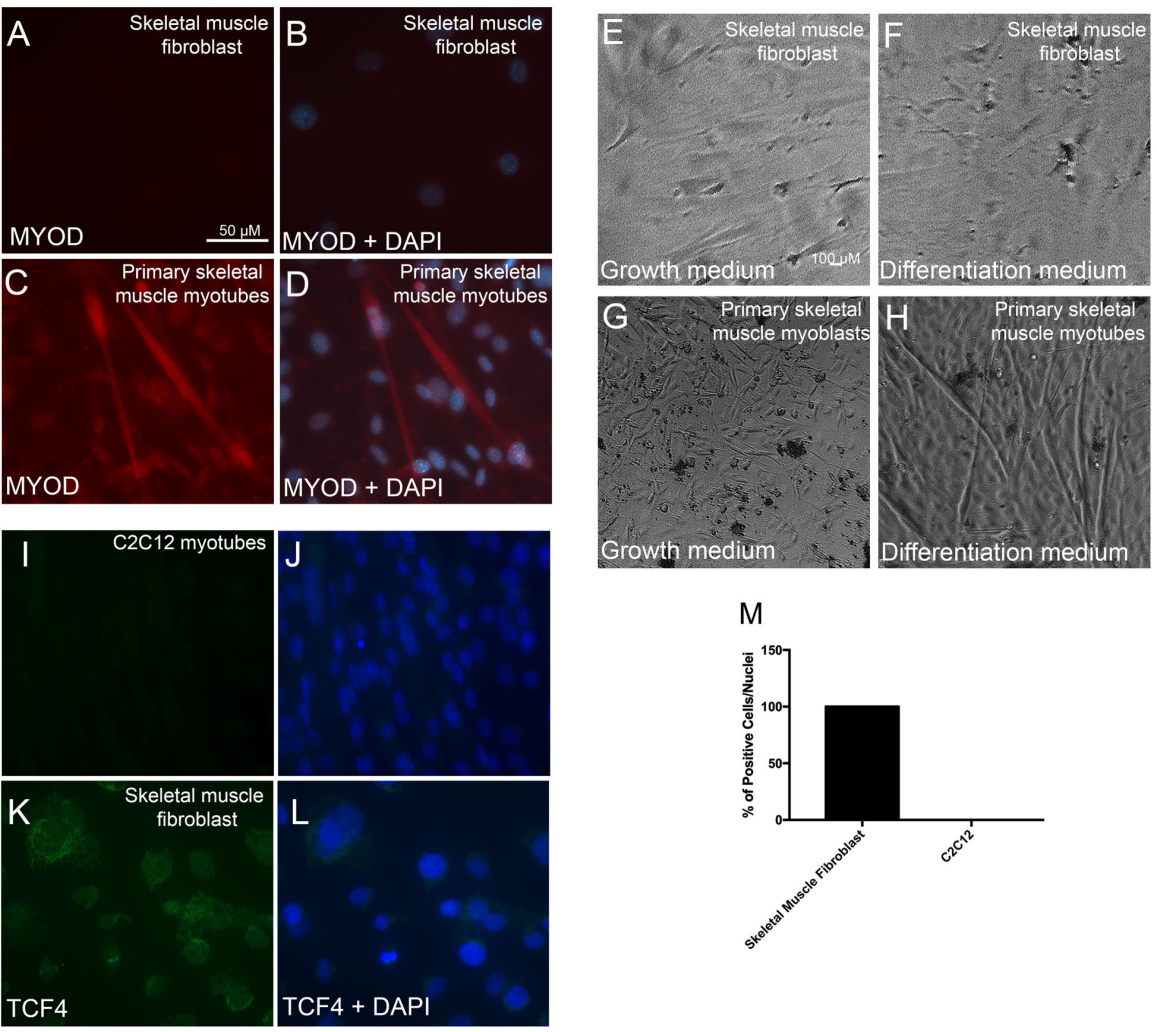
Validation of fibroblast culture. Immunofluorescence for MyoD in skeletal muscle fibroblasts (**A**, **B**) and primary skeletal muscle myotubes as a positive control for MyoD labelling (**C**, **D**) 2 days after differentiation medium (2% HS). Phase contrast microscope image from skeletal muscle fibroblasts submitted to either growth medium with 80-90% confluence (**E**) or differentiation medium for 2 days (**F**). Myoblasts were also submitted to either growth medium with 80-90% confluence (**G**) or differentiation medium for 2 days (**H**) as a positive control. Immunofluorescence for TCF4 in C2C12 myotubes differentiated for 2 days (**I** and **J**) and in skeletal muscle fibroblasts (**K** and **L**). **M**: Percentage of TCF4-positive cells. Images were taken at high (**A**-**D** and **I**-**L**, 400×; bar, 50 µM) and low (**E**-**H**, 40×; bar, 100 µM) magnifications.

Our first aim was to confirm the degree of fibroblast pureness in our cultures. Accordingly, we observed no expression of the myoblast myogenic marker MyoD (Figure 1A and B). Furthermore, as a positive control, we detected MyoD expression in the cells from P0 myoblast-enriched pool of primary myoblasts (Figure 1C and D). Finally, we observed no formation of myotubes in our skeletal muscle fibroblasts (Figure 1E and F), nonetheless, as expected, myotube formation was confirmed when using differentiation medium in the cells from P0 myoblast-enriched primary myoblasts pool (Figure 1G and H). In order to confirm the fibroblast purity, we aimed to detect TCF4 (transcription factor 7-like 2, Tcf7L2-Genome Mouse Browser), which is considered a hallmark of the fibroblast phenotype (5,25). The analysis showed that 100% of skeletal muscle fibroblasts were positive to TCF4 (Figure 1K, L and M) while, as expected, C2C12 showed no labeling to TCF4 (Figure 1I and J) confirming that skeletal muscle fibroblasts used in this study do not contain myogenic cells.

### MuRF2 was expressed in fibroblasts

**Figure 2.**
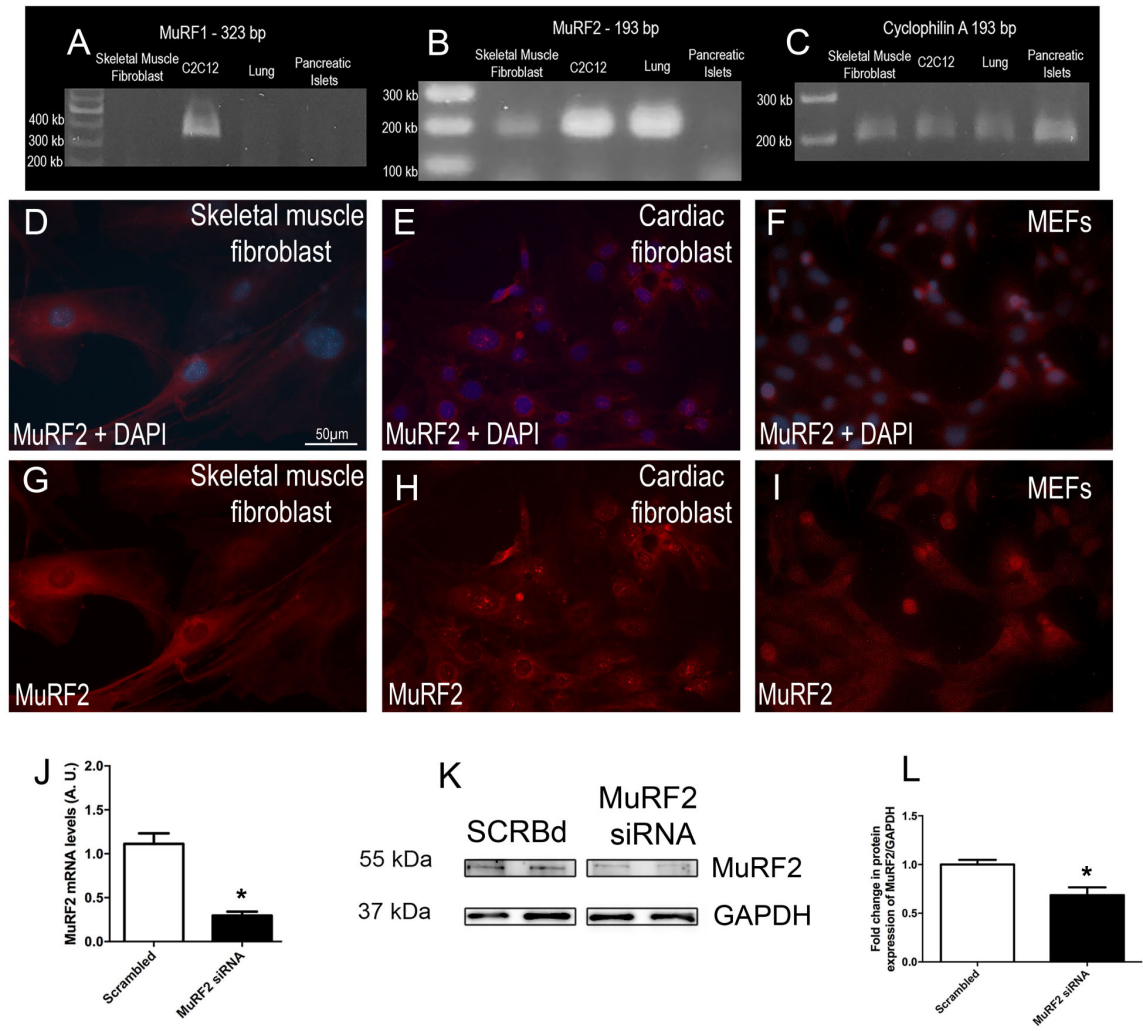
MuRF2 is expressed in fibroblasts. Semi-quantitative PCR for MuRF1 (**A**) and MuRF2 (**B**), and cyclophilin as a housekeeping gene (**C**) in skeletal muscle fibroblasts, C2C12, lung, and pancreatic islets. Immunofluorescence for MuRF2 in skeletal muscle fibroblasts (**D** and **G**), cardiac fibroblasts (**E** and **H**), and mouse embryonic fibroblasts (MEFs) (**F** and **I**). **J**: Real-time PCR in skeletal muscle fibroblasts treated with scrambled (SCRBd) or MuRF2 siRNA. **K**: Representative immunoblots for MuRF2 and GAPDH in skeletal muscle fibroblasts treated with SCRBd or MuRF2 siRNA. The images belong to the same membrane. **L**: Relative band intensities were determined by densitometry. Scale bar, 50 µM (**D-I**). Data are reported as means±SE. *P<0.05 *vs* SCRBd (Student’s *t*-test).

To verify expression of MuRF1 and MuRF2 in skeletal muscle fibroblasts, we performed a semi-quantitative PCR using C2C12 as a positive control, lung and pancreatic islets as negative controls, using primers for MuRF1 and MuRF2, generating amplicons of 323 and 193 bp, respectively. We observed that skeletal muscle fibroblasts did not express MuRF1 as well as lung and pancreatic islets (Figure 2A). Cyclophilin was used as a housekeeping gene (Figure 2C). These results were expected as previously shown (17). On the other hand, we found MuRF2 expression in skeletal muscle fibroblasts, along with C2C12 (a purified skeletal muscle cell line) in mouse lung tissue (Figure 2B). Then, we analyzed the cell localization of MuRF2 in three different fibroblast cells by immunofluorescence. Our results showed that MuRF2 was scattered within the cytoplasm, with perinuclear accumulation in skeletal muscle fibroblasts (Figure 2D and G). In primary cardiac fibroblast cells, similarly, we found MuRF2 labeling scattered within the cytoplasm, with perinuclear accumulation (Figure 2E and H). Moreover, when using a commonly used fibroblast cell line (MEFs), we identified a strong labeling in MEFs nucleus, by immunofluorescence for MuRF2 (Figure 2F and I). Thus, our immunofluorescence analysis showed that MuRF2 was present in fibroblasts of different tissue sources.

In order to confirm the MuRF2 expression in skeletal muscle fibroblasts, we knocked-down MuRF2 using siRNA (MuRF2 siRNA) and found a decrease in both mRNA and protein expression compared to the scrambled (SCRBd) group (Figure 2J, K, and L).

### Knock-down of MuRF2 impaired skeletal muscle fibroblast wound healing capacity

**Figure 3.**
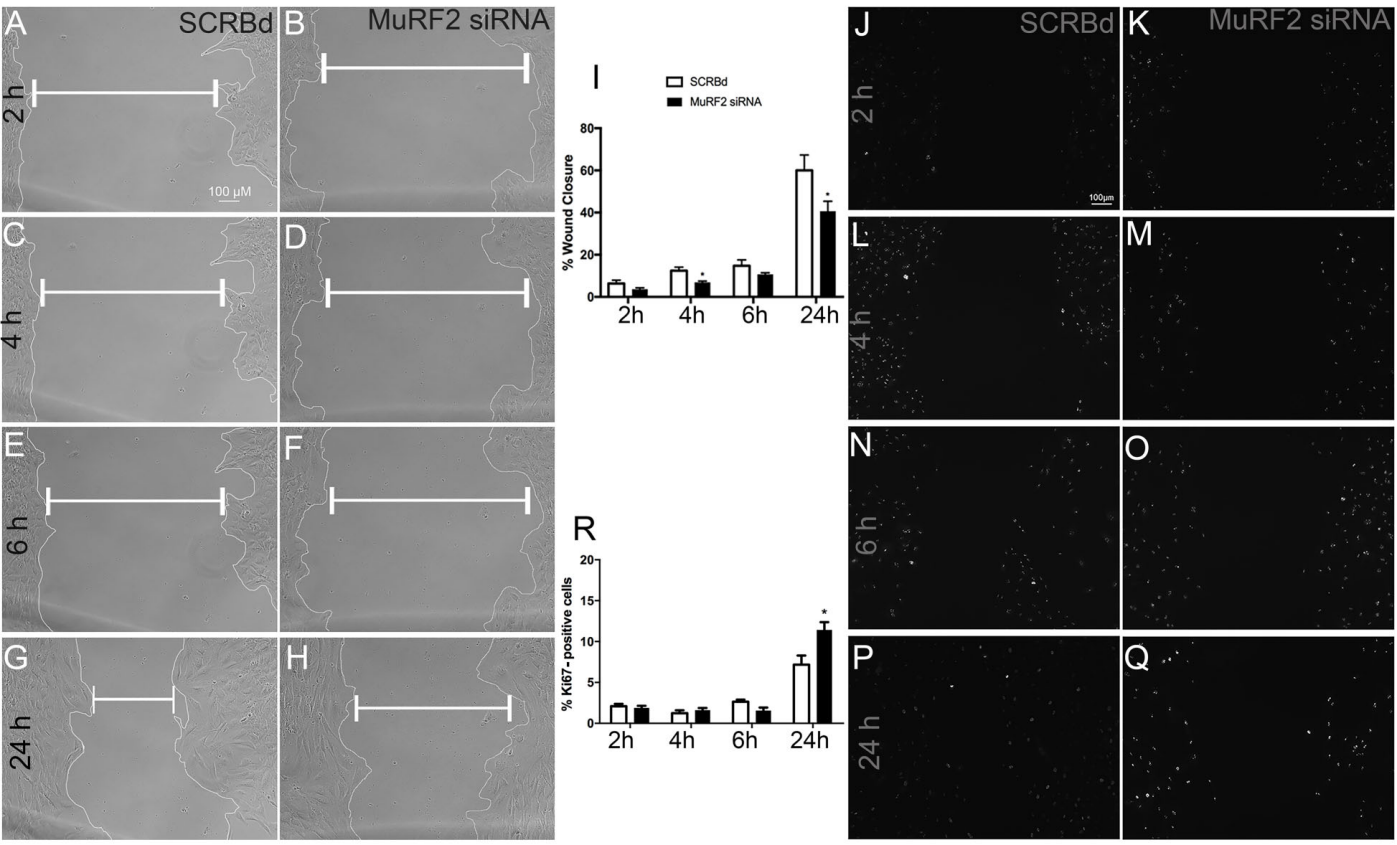
Knock-down of MuRF2 decreased skeletal muscle fibroblast wound healing capacity. Phase contrast microscope image from skeletal muscle fibroblasts in wound healing assay at different time points (2, 4, 6, and 24 h). Skeletal muscle fibroblasts treated with scrambled (SCRBd) (**A**, **C**, **E**, and **G**) and with MuRF2 siRNA (**B**, **D**, **F**, and **H**); **I**: Percentage of wound closure obtained from the phase contrast microscope. Ki67-positive nuclei in skeletal muscle fibroblasts submitted to wound healing assay at 2, 4, 6 and 24 h (**J**–**Q**). Skeletal muscle fibroblasts treated with SCRBd (**J**, **L**, **N,** and **P**) and with MuRF2 siRNA (**K**, **M**, **O**, and **Q**). **R**: Percentage of Ki67-positive cells. Data are reported as means±SE. *P<0.05 *vs* SCRBd (*t*-test). Scale bar, 100 µM.

To gain insight into the role of the E3 ligase MuRF2 in skeletal muscle fibroblast, we conducted wound healing assays using MuRF2 siRNA. The knock-down of MuRF2 caused a significant decrease in the fibroblast migration capacity after 4 and 24 h (Figure 3D, H, and I).

Next, we addressed whether the silencing of MuRF2 induced changes in the proliferation of skeletal muscle fibroblasts using Ki67 immunolabeling (Figure 3J-R). Our results showed that MuRF2 siRNA caused a significant increase in Ki67-positive nuclei abundance after 24 h (Figure 3Q and R).

### Knock-down of MuRF2 in skeletal muscle fibroblast impaired lamellipodia formation

**Figure 4.**
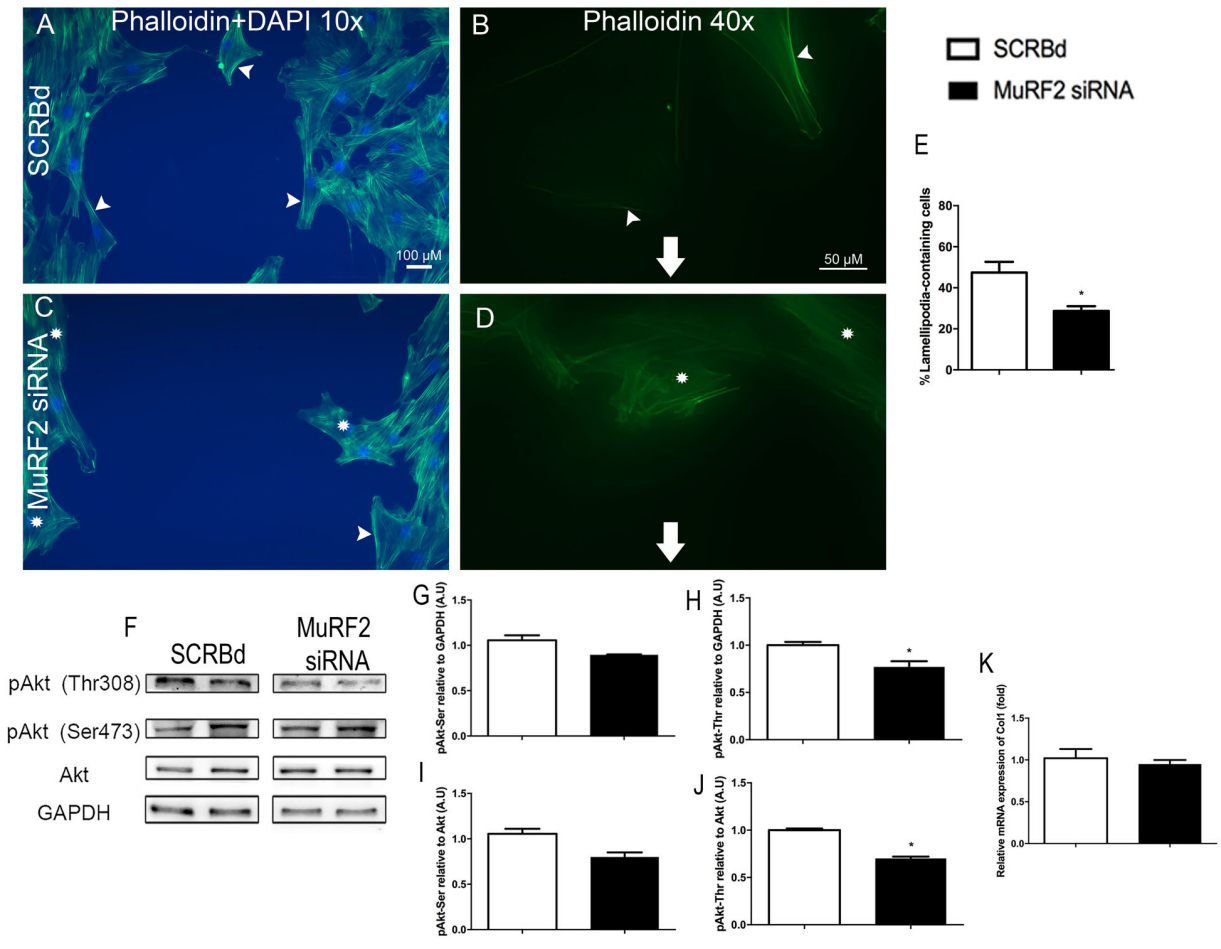
Knock-down of MuRF2 in skeletal muscle fibroblasts decreased lamellipodia formation. Staining for phalloidin and DAPI 24 h after the onset of wound healing assays at low (**A** and **C**, 100×; bar, 100 µM) and high (**B** and **D**, 400×; bar, 50 µM) magnifications; Skeletal muscle fibroblasts treated with scrambled (SCRBd) (**A** and **B**) and with MuRF2 siRNA (**C** and **D**). Arrowheads indicate actin stress fibers, asterisks indicate misfolded actin cytoskeleton, and arrows indicate cell migration direction. **E**: Quantification of lamellipodia-containing cells. **F**: Representative immunoblots of pAkt Ser473, pAkt Thr308, total Akt, and GAPDH in skeletal muscle fibroblasts. The images belong to the same membrane. **G**-**J**: Densitometry from western blots. **K**: Real-time PCR for collagen 1 (Col1) in skeletal muscle fibroblast. Data are reported means±SE. *P<0.05 *vs* SCRBd (*t*-test).

Given that MuRF2 is developmentally associated with microtubules (26), we hypothesized that lack of MuRF2 could lead to cytoskeleton disorganization. Therefore, we performed wound healing assays after 12 h of MuRF2 knock-down. Twenty-four hours after the onset of wound healing assays, the incidence of lamellipodia was analyzed using fluorescent dye conjugated to phalloidin-Alexa Fluor 488 to detect polymerized actin. Figure 4A, B, and E shows the normal distribution of actin fibers in the SCRBd group, in which the lamellipodia are clearly visible. When MuRF2 was knocked-down, skeletal muscle fibroblasts exhibited a reduction in lamellipodia formation (∼50% reduction, P<0.05) (Figure 4C, D, and E), compromising the organization of the cytoskeleton that is essential for cell migration (27).

Because the Akt Ser473 and Akt Thr308 residues are important in cell migration (12,24), we decided to address their level of phosphorylation in MuRF2 knocked-down fibroblasts using specific antibodies. Even though we did not find a difference in Akt phosphorylation level at the Ser473 residue, we observed a strong reduction in Akt phosphorylation level at the Thr308 residue after MuRF2 siRNA compared to the scrambled group (Figure 4F, H, and J).

Since collagen 1 is abundantly expressed and plays a key role in skeletal muscle tissue (28), we analyzed whether MuRF2 siRNA could modulate collagen mRNA levels. Our results showed that decreased MuRF2 expression in skeletal muscle fibroblasts did not cause alterations in collagen mRNA levels (Figure 4K).

## Discussion

In the present study, we showed that MuRF2 plays an important role in skeletal muscle fibroblast function, notably acting on migration capacity. We have used a cell culture model of primary skeletal muscle fibroblasts and initially it was essential to certify that these cultures were devoid of myoblasts. We therefore labeled these cultures with MyoD (a myoblast marker) (29) and TCF4 (a fibroblast marker) (5). By using these markers, we assured that the cultures utilized were fibroblast enriched and contained no myoblasts. In addition, when a myogenic stimulus (2% horse serum) was applied to these cultures, we observed no myotube formation, further reassuring the absence of myoblasts. Finally, the cells used in the present study exhibited a flattened and stellate format, typically recognized as morphological characteristics of fibroblasts (30).

MuRF2 has been recognized as an important player in a variety of processes, such as in the ubiquitin proteasome system and autophagy, which seems to depend mainly upon poly-ubiquitination (31). Also, MuRF2 has been shown to be involved in the stabilization of skeletal muscle fiber structural proteins. For example, Pizon et al. reported that MuRF2 interacts with microtubules and this complex subsequently associates with sarcomeric myosin. Their results indicate that MuRF2 is necessary for proper sarcomere assembling (32). Another work established that MuRF2 is involved in sarcomeric stabilization also via intermediate filaments. Antisense oligonucleotide MuRF2 knock-down perturbed the stability of desmin and vimentin (18). Combined, these data strongly suggest that MuRF2 works as a cytoskeleton organizer, exerting an important role in sarcomeric structure assembly and stabilization. Interestingly, a subsequent study (33) showed that MuRF2 and MuRF3 deletion in mice causes severe skeletal muscle histological alterations, including sarcomeric disarrays, reinforcing the notion that MuRF2 can be involved in cytoskeleton organization. More recently, MuRF2 was also shown to be a skeletal muscle tissue organizer in mice with MuRF1 and MuRF2 deletion (21). In spite of these data clearly establishing a connection between MuRF2 and the skeletal muscle fiber cytoskeleton, no studies had so far approached fibroblasts, which are cells extremely dependent upon their cytoskeleton to function.

MuRF2 was initially described as being expressed in skeletal and cardiac muscles along with its counterpart MuRF1 (16). Subsequent studies showed the presence of MuRF2 also in non-muscular tissues, such as a low expression of MuRF2 in the liver of pigs (34). More recently, Bian and coworkers found MuRF2 expression in several tissues, including lung, spleen, brain, liver, and kidney (35). Therefore, it is well accepted that MuRF2 is not restricted to muscle tissue, nonetheless, few studies are available addressing the localization and role of MuRF2 in specific cell types. Here, we showed that skeletal muscle fibroblasts can express MuRF2, in addition to the expected expression observed in the skeletal muscle fiber (21). Importantly, herein, MuRF2 cellular localization was predominantly found in the cytoplasm in skeletal muscle fibroblasts, whereas in cardiac fibroblasts, additional intense punctiform nuclear labelling was also observed. These results indicated that MuRF2 might exert differential effects depending upon which tissue fibroblasts are located. Possible mechanisms include poly-ubiquitination and subsequent degradation of specific sets of target proteins, or mono-ubiquitination, leading to changes in their activities. It would be interesting in forthcoming studies to comparatively address the impact of MuRF2 deletion in the migration capacity of fibroblasts from distinct sources.

Herein, we showed that MuRF2 silencing decreased the migratory activity of fibroblasts. In addition, we have shown that MuRF2 silencing increased cell proliferation by KI67 immunolabeling (Figure 3). We envisioned that those results combined might mean that MuRF2 could be involved in limiting cell cycle, maybe driving the cell to a more differentiated phenotype, which in turn could favor organization of cytoskeleton and consequently more migratory capacity.

In addition to skeletal muscle and cardiac fibroblasts, we also addressed mouse embryonic fibroblasts (MEFs). In MEF cells, we observed a widespread localization of MuRF2, including in the cytoplasm and the nucleus. Considering that they are embryonic fibroblasts, we interpreted that MuRF2 is an early expressed gene, participating in initial stages of cytoskeleton stability. Although currently the biological meaning of those differences in cellular localization is not clear, it could be related to key functions of those cells, such as migration and secretory activity. The control of such important functions depends upon intracellular pathways and some of them have been well described. For example, platelet-derived growth factors are central chemosensory regulators of the migratory capacity in wound healing (36). In addition, PI(3,4,5)P_3_-mediated reorganization of actin cytoskeleton can proceed by activation of Akt, which physically interacts with actin. In fact, Rahman and coworkers showed that Akt activity is crucial to the formation of actin filaments at the edge of fibroblasts during the migration process (12). Then, to address related underlying mechanisms, we investigated Akt activity, a key cytoskeleton regulator. MuRF2 siRNA caused a robust reduction in Akt phosphorylation of Thr308, strongly suggesting that MuRF2 is necessary to sustain the actin cytoskeleton functionality via maintenance of Akt activity. Our results also indicated that Akt Ser473 phosphorylation was not altered by MuRF2 silencing. Since it has been shown that Thr308 phosphorylation is accomplished by PDK1 (37), we envisioned that effects of MuRF2 upon cytoskeleton remodeling depend, at least part, on PDK1 preserved activity. The precise mechanisms involved are currently not known, nonetheless it is possible that MuRF2 could poly-ubiquitinate and degrade an inhibitor of PDK1. Indeed, it is known that the protein Claudin-18 can inhibit PDK1 phosphorylation (38), therefore Claudin-18 could be considered a potential candidate for such role. Alternatively, MuRF2 could mono-ubiquitinate PDK1 keeping enzyme activity at appropriate levels. Actually, it has been shown that MuRF2 can mono-ubiquitinate PPARα and PPARγ maintaining their activity and preserving cardiac function (39). Importantly, in the present study, we did see a correlation between decreased Akt activity and changes in cytoskeleton since MuRF2 siRNA caused about 50% decrease in lamellipodia formation. It is largely known that lamellipodia is a major actin cytoskeleton organization process involved in cell migration (12,27).

Regarding ECM production by fibroblasts, it is known that TGF-β1 binds to its membrane receptor, which in turn activates a series of phosphorylation, promoting phosphorylation of the Smad2/3 complex. This complex also acts together with Smad4, regulating transcription of extracellular matrix proteins (40). In order to approach ECM production, we measured mRNA levels of collagen 1 and our results showed no difference in collagen 1 mRNA in MuRF2 knocked-down skeletal muscle fibroblasts, suggesting, overall, that this E3 ligase plays an important role in fibroblast migration but not in ECM synthesis.

In conclusion, this report suggests that MuRF2 played an important role in the functional capacity of skeletal muscle fibroblasts by reducing the activation of Akt and consequently compromising the organization of the cytoskeleton. These findings provided novel insights into the regulation of skeletal muscle fibroblasts, with possible implications for conditions in which intense ECM remodeling occurs, such as during skeletal muscle regeneration, where increased migratory activity of fibroblasts is required.
